# SHOULD PEDIATRICIANS INVESTIGATE THE SYMPTOMS OF OBSESSIVE-COMPULSIVE DISORDER IN CHILDREN WITH FEEDING DIFFICULTIES?

**DOI:** 10.1590/1984-0462/;2019;37;1;00010

**Published:** 2018-10-29

**Authors:** Ana Beatriz Bozzini, Gabriela Malzyner, Priscila Maximino, Rachel Helena Vieira Machado, Claudia de Cassia Ramos, Letícia Ribeiro, Mauro Fisberg

**Affiliations:** a Instituto PENSI - Hospital Infantil Sabará, São Paulo, SP, Brasil.

**Keywords:** Feeding and eating disorders, Obsessive-compulsive disorder, Child psychiatry, Child, Adolescent, Transtorno da alimentação e da ingestão de alimentos, Transtorno obsessivo-compulsivo, Psiquiatria infantil, Crianças, Adolescente

## Abstract

**Objective:**

To review current evidence on the relationship between obsessive-compulsive disorder and feeding difficulties.

**Methods:**

Review the *Science Direct* and *PubMed* databases between 2007 and 2017 in English, Portuguese and Spanish. The search terms, used in association, were “obsessive compulsive disorder” and “picky eating/feeding difficulty”. Cohort, case control and cross sectional studies were included that analyzed children, adolescents and/or adults of any sample size from any country in the world. Opinion articles were excluded.

**Results:**

Around 245 articles were selected, and only 4 were included in this review, according to previous criteria. Results from the studies essentially described that there is indeed a difference in “picky” behaviors between subjects with and without obsessive-compulsive disorder. Patients with obsessive-compulsive disorder tend to have exacerbated symptoms of disgust, anxiety and a higher eating behavior inflexibility score.

**Conclusions:**

Obsessive-compulsive disorder and feeding difficulties patients share common symptoms. The present study alerts health professionals who follow patients with feeding difficulties as to the importance of investigating possible psychiatric comorbidities.

## RESUMO

**Objetivo:**

Revisar evidências atuais da relação entre transtorno obsessivo compulsivo e dificuldades alimentares.

**Métodos:**

Revisão das bases *Science Direct* e PubMed no período entre 2007 e 2017 em inglês, português e espanhol, com os termos em associação “transtorno obsessivo compulsivo” e “*picky eating*/dificuldade alimentar”. Foram selecionados apenas estudos de coorte, caso controle ou transversal, realizados em qualquer país, com crianças, adolescentes e/ou adultos e de qualquer tamanho amostral. Foram excluídos os artigos de opinião.

**Resultados:**

Cerca de 245 artigos foram selecionados e apenas 4 foram incluídos no estudo, segundo critérios de seleção. Os trabalhos descrevem essencialmente que há diferença no comportamento seletivo entre os sujeitos com e sem transtorno obsessivo compulsivo, com tendência para exacerbação de sintomas como nojo, ansiedade e escore de inflexibilidade de comportamento alimentar nos pacientes com esse transtorno.

**Conclusões:**

Existem sintomas compartilhados entre transtorno obsessivo compulsivo e dificuldade alimentar. O estudo alerta aos profissionais que acompanham pacientes com dificuldades alimentares para a importância da investigação de possíveis comorbidades psiquiátricas.

## INTRODUCTION

Obsessive compulsive disorder (OCD) is known as a disease that is characterized by patterns of repetition, symptoms of anxiety and inflexible behaviors.^1^ It was previously classified as an anxiety disorder and is now part of the “OCD and related disorders” group in the Diagnostic and Statistical Manual of Mental Disorders (DSM-5).^2^ Even though it is rare in childhood, worldwide prevalence ranges from 0.25 to 4% among children and adolescents. British data show a prevalence of under diagnosed and under-treated OCD of 0.25% in a sample of children and adolescents between 5 and 15 years old, and this prevalence increases in the older age groups. Other recent studies have shown a subclinical OCD rate in children of 10%.^3^ In Brazil, there is little epidemiological data and the prevalence is 0.7 to 2.1%, according to data from a multi-center study in São Paulo, Brasília and Porto Alegre.^4^ These children are described as belonging mainly to lower socioeconomic classes and they have lower levels of schooling.^1^

The etiology of OCD remains poorly understood, although empirical evidence corroborates the hypothesis of a genetic component at its origin. Its genetic inheritance is considered to be polygenic, as it exerts a relatively small effect on the phenotype on its own. In particular, genes from the serotonergic pathway, the dopaminergic system, and the glutamatergic system, seem to influence the development of OCD,^5^ as they act on several different mechanisms, from the change in serotonin receptors to the transport of brain stimuli. The diagnostic criteria for OCD in children are very similar to those of adults. However, children are less aware of their obsessive compulsive behaviors and it is important to differentiate the actual compulsions from the rituals of their routine, which typically include sleeping, eating, bathing, and other daily activities.^6^

It is known that the presence of OCD during childhood increases the chance of other psychiatric disorders in adulthood, and that there is a strong correlation between OCD and family history. In a Brazilian study, Ferrão et al*.* ^7^ show that patients with OCD and a positive family history present the onset of symptoms earlier, present them with a greater severity and with a greater therapeutic complexity, not to mention there are more patients that practice hoarding. In childhood, the peak incidence for OCD is at age 11, and approximately 50% of adults with OCD showed symptoms before the age of 18. Although it also manifests in younger children, it is difficult to definitively diagnose OCD in very small children, since they are in a phase of intense dynamic psycho-emotional development and are not completely exposed to the demands of the external world. In addition, the window of opportunity to modify psychological symptoms in childhood is mainly from 0 to 3 years old due to the cerebral plasticity that exists in early childhood.^8^ However, the earlier the disorder sets in, the greater the chance of comorbidity and severity, if no intervention is performed. Therefore, early diagnosis is of great importance. The main comorbidities of childhood OCD are autism, attention deficit hyperactivity disorder (ADHD), oppositional defiant disorder, disruptive behaviors, phobias and anxiety disorders.^9^

Symptoms of this disease may improve or worsen throughout treatment, but generally they tend to follow a chronic course, causing significant functional impairment in a number of domains including home, school, and the social environment.^6^ As the symptoms of the disease lead to impairments in various aspects of the patient’s life, some symptoms of OCD may be reflected in eating practices, regardless of the age group in which this disorder occurs. In 2015, Kauer et al. showed that adults with food selectivity have higher OCD and depression scores than non-selective ones, concluding that there is a need to go deeper into the subject in order to better understand the behavior of selective patients.^10^

Eating difficulties (EDs) in childhood are defined as any disagreement or difficulty between caregiver and child with regard to food. They include cases of selectivity, food phobias, organic disorders or even misinterpretation of the parents. Eating difficulties are a very frequent complaint in pediatric clinics, with a worldwide prevalence of 30%.^11^

Many of these eating difficulties show symptoms of extreme rigidity and inflexibility with regard to eating behavior, resembling OCD symptoms. However, the literature regarding OCD and inflexible behaviors and their relationships with selectivity and other EDs is scarce. Just as there are studies that associate symptoms of OCD with food selectivity in the adult population, research that involves children should also be conducted in order to seek associations between both, since refusal to eat is one of the most frequent complaints heard in pediatric clinics. Are comorbidities possible? Are EDs some of the positive predictors of OCD? The objective of this study was, thus, to review current evidence with regard to the possible relationships between EDs and OCD.

## METHOD

This study reviewed *Science Direct* and *PubMed* bibliographic databases in the period between 2007 and 2017, in English, Portuguese and Spanish. The search terms were used in association, according to the Boolean operator “and”, and between “obsessive compulsive disorder” and “picky eating / feeding difficulty”. The inclusion criteria were based on the selection of studies that aimed at demonstrating OCD and EDs comorbidity and/or discussing the relationship between OCD and EDs symptoms. We selected only cohort, case control or cross-sectional studies that were conducted in any country, and analyzed children and/or adolescents in any sample size, and in populations that did or did not have comorbidities. Given the scarcity of work with pediatric populations, studies with adults were included in the review. Opinion articles were excluded.

The selection process of the studies followed these steps:

1. reading the titles and summaries of the articles found;
2. excluding works outside the selection criteria, and duplicates;
3. excluding studies with a research focus that was incompatible with the objectives of this study;
4. excluding articles where the full text was not available.

## RESULTS

**Figure 1:**
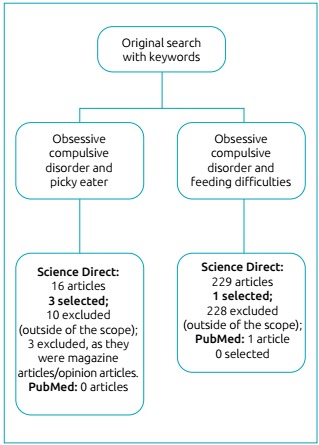
Flow chart of the article selection process.

No articles were found in *PubMed* with the descriptors “obsessive compulsive disorder and picky eater”. In the *Science Direct* network, 16 articles were found, of which 3 were selected. When we used the descriptors “obsessive compulsive disorder and feeding difficulties” in *PubMed*, we found only 1 article published in the past 10 years and it did not meet the inclusion criteria for this study. In *Science Direct*, a total of 229 articles were found. Of these, only one was selected, since the others did not fit the objectives or did not meet the inclusion criteria. Of the total articles found, only four were selected, according to the inclusion criteria. The results of the selection process are described in Figure 1.

The evaluated studies showed that selective patients have significantly more OCD symptoms than non-selective ones, and they show a tendency to have exacerbated symptoms such as disgust, anxiety and a high food behavior inflexibility score. The detailed results are described in Table 1.

**Table 1:** Description and results of the selected studies.

Reference	Objective	Design and population	Variables used	Results
2015, Kauer et al*.* ^10^ (United States)	Compare psychosocial and behavioral behaviors between selective and nonselective adults.	Cross-sectional (n=489 adults).	Feeding questionnaire, self-diagnosis of selectivity, OCD, depression, feeding disorder, sensorial and neophobia symptoms.	The selective adults presented higher levels of OCD symptoms (sensitivity to disgust and food neophobia) and were more likely to be included in the clinical range for symptoms of depression (p<0.01).
2016, Zickgraf et al*.* ^12^ (United States)	Verify restrictive feeding behavior in adults.	Cross-sectional (n=139 adults).	Questionnaires with socioeconomic and feeding information (feeding variety and inflexible behavior).	The inflexibility behavior score was higher in selective individuals in relation to nonselective individuals (p<0.01).
2016, Zickgraf et al*.* ^13^ (United States)	Identify comorbidities in selective adults.	Cross-sectional (n=332 nonselective adults *versus* n=81 selective adults).	Comparison between the two groups with regard to OCD, stress, loss of quality of life, neophobia, sensorial, ARFID, and inflexible behavior symptoms.	Selective patients with ARFID more frequently had symptoms of OCD than patients that were only selective (p<0.001).
2015, Johnson et al*.* ^14^ (United States)	Compare anxiety symptoms in autistic children.	Cross-sectional (n=118 children between 2 and 6 years old).	Questionnaires about sleep, anxiety and feeding.	Anxiety symptoms in children increased the chance for feeding difficulties (p<0.001).

OCD: Obsessive compulsive disorder; ARFID: *avoidant/restrictive food intake disorder*.

## DISCUSSSION

The results demonstrate the scarcity of publications on the subject and the need for more studies regarding OCD and EDs. Of all the articles found, only two related symptoms or characteristics of OCD with EDs, and two others dealt mainly with symptoms of anxiety and rigid behavior. Most of the articles found dealt only with OCD or its relationship with eating disorders (mainly anorexia nervosa), and not with EDs. In addition, most of the studies were conducted with an adult population. The only article found with a pediatric population evaluated autistic children, which in itself represents a bias in the analyses.

The articles found show that there are some common characteristics in the behavior of selective and OCD patients: the selective ones rejected food that had been mixed or previously touched by someone, food that had touched a plate, and additionally, they were repulsed by certain textures. Furthermore, selective patients had significantly more inflexibility than non-selective ones,^10^ and the compulsive and obsessive symptoms of selective ones were often not exclusively related to food. A clinical study that attempted to map out a descriptive profile of 33 selective children between 4 and 14 years of age demonstrated that selective children suffer from obsessive compulsive symptoms and anxiety that may or may not be related to food. In addition, they often had difficulties at school and in social situations.^15^

It is known that the most frequent symptoms of OCD in childhood are: fear of contamination, fear of injuring themselves or others, sexual and symmetrical obsessions, compulsions with washing, checking, repeating, counting, ordering/arranging, and tic-like compulsions, all of which generate suffering for the child.^16^ On the other hand, the behavior of patients with EDs with regard to food varies from physical reactions to even making meticulous choices, counting grains, choosing packaging, repulsion from touching, feeling or smelling.^11^ In the population attended at the Center for Food Difficulties of the Teaching and Research in Childhood Health Institute (*Centro de Dificuldades Alimentares do Instituto Pesquisa e Ensino em Saúde Infantil* - CDA - Instituto PENSI),^17^ it was observed that some of the common behaviors between OCD and eating disorders may be present in the selective patients and patients with other EDs, such as: not getting dirty with food; intolerance of mixing different foods, separating food by color, and food’s appearance or texture; intolerance of changing the brand packaging of a particular product; and not accepting modifications in the way the food is presented at the time of a meal. In general, these patients did not make their behavior more flexible with regard to their own food choices, even if they were quite restrictive and selective.

Perhaps for this reason, studies such as that of Zickgraf et al*.* ^13^ separate the selective patients into two groups: those with a comorbidity (called restrictive or avoidant) and purely selective patients. This study shows that the selective group with the so-called “restrictive” comorbidity (ARFID - avoidant/restrictive food intake disorder) presents symptoms along with those with OCD, including inflexibility and loss of quality of life related to feeding more often than the selective-only ones, without a comorbidity (p <0.001). Likewise, the clinical experience of the CDA of the PENSI Institute describes a trend towards a higher restrictive food acceptance pattern (acceptance of less than 15 foods) in children with associated comorbidities. “Restrictive comorbidity” is a new diagnosis and can occur at any age, leading to weight loss, nutritional deficiencies, the need for supplementations, or psychosocial impairments, but has no psychopathological basis for concern about body or weight, unlike eating disorders such as anorexia and bulimia.

The timing of the meal for patients with EDs ended up being a source of anxiety for the children and their families and, according to Timimi et al*.* ^15^, a history of depression in at least one of the parents was found in one third of the evaluated cases. It is important to question whether the anxiety symptoms themselves, as in the case of OCD, do not lead to EDs appearing in pediatric clinics as the main complaint and symptom. A study conducted in Philadelphia in 2008^14^ found that anxious children had a higher chance of EDs, as well as sleep disorders. In this regard, EDs could occur due to some symptom of anxiety rather than vice versa, and therefore, the pediatrician who is dealing with a food complaint must understand whether the symptom of anxiety is the main disruptor of the different areas of the patient’s life, including food.

Another question with regard to childhood anxiety symptoms is whether they are a predictive trait of future disorders. Among eating disorders, it is known that OCD and anorexia nervosa, for example, are frequent comorbidities, and many researchers are searching for common genetic and/or biochemical etiology.^18^ ^,^ ^19^ ^,^ ^20^ What can be inferred so far is that selective patients are more rigid in their food behavior. However, it is not possible to affirm relationships based on the development of OCD itself.

Environmental factors also play an important role in affecting the disorder, although very little is known about them. For example, a longitudinal study mentions social isolation, physical abuse and negative emotions as specific diagnostic predictors.^21^ For adult patients, a recent retrospective study found evidence for an association between adverse experiences (traumas) in childhood and OCD.^21^ A national study comparing OCD patients with a positive history for trauma versus a history with no trauma found no statistical difference in response to treatment.^22^ Many EDs may be associated with trauma, such as threats made around eating, hospitalizations in Intensive Care Units (ICUs), or excessive physical manipulation. It is worth questioning, then, if the selective or phobic behavior of some children with regard to food originates from some trauma or a misunderstanding of what certain foods represent.

In parallel, the environmental component is strictly related to the development of EDs in childhood. The so-called “responsive caregiving” method (a set of caregiver behaviors that address attention and interest in the child care process with regard to various health aspects) influences the formation of eating habits and the nutritional status of the child from an early age, and encourages the appropriate development of social skills, learning, self-esteem, autonomy and independence in all spheres of development.^23^

Clinically, the profile of the relationship between caregivers and children with regard to ED complaints is typically described as nonresponsive, which may cause family’s to fall out of balance. The clinical experience of the CDA from the PENSI Institute describes high rates of nonresponsive care among mothers of children with complaints of EDs. Such behaviors include: offering meals in inappropriate environments and in uncomfortable body postures, using physical coercion and distractions to offer food, not eating meals together, and disrespecting the child’s signs of hunger. A family with this kind of profile may exacerbate the eating symptoms of these patients, or even cause them. Just as the inflexible and rigid behaviors of the OCD patient directly affect their quality of life, selective patients may have a loss in quality of life due to the same behaviors in relation to food.

Therefore, more studies are necessary in order to verify the psychic and neurobiological relationships between selectivity and OCD patterns, in order to avoid under diagnosing and to invest in new forms of treatment. It is important that doctors who receive many patients with EDs, especially pediatricians, are aware of the symptoms of OCD, and if necessary, recommend the patient for a psychiatric evaluation.

There are common symptoms between OCD and EDs in adults, such as inflexibility; anxiety and rigid behavior; and food choices based on specific characteristics such as color, smell and textures. However, the literature is still minimal, and more cohort studies are needed to identify shared symptoms and to establish significant relationships between these two conditions in childhood. As the complaint of EDs is very frequent in childhood, it is up to medical professionals to investigate the various aspects of the life of patients with this type of difficulty, not just restricting themselves to food complaints, in order to avoid the under diagnosis of possible psychiatric comorbidities.
